# Label‐free single cell proteomics utilizing ultrafast LC and MS instrumentation: A valuable complementary technique to multiplexing

**DOI:** 10.1002/pmic.202200162

**Published:** 2023-03-01

**Authors:** Manuel Matzinger, Rupert L. Mayer, Karl Mechtler

**Affiliations:** ^1^ Research Institute of Molecular Pathology (IMP) Vienna BioCenter Vienna Austria; ^2^ Gregor Mendel Institute of Molecular Plant Biology (GMI), Austrian Academy of Sciences Vienna BioCenter (VBC) Vienna Austria; ^3^ Institute of Molecular Biotechnology (IMBA), Austrian Academy of Sciences Vienna BioCenter (VBC) Vienna Austria

**Keywords:** bioinformatics, cell biology, label‐free, quantification, technology

## Abstract

The ability to map a proteomic fingerprint to transcriptomic data would master the understanding of how gene expression translates into actual phenotype. In contrast to nucleic acid sequencing, in vitro protein amplification is impossible and no single cell proteomic workflow has been established as gold standard yet. Advances in microfluidic sample preparation, multi‐dimensional sample separation, sophisticated data acquisition strategies, and intelligent data analysis algorithms have resulted in major improvements to successfully analyze such tiny sample amounts with steadily boosted performance. However, among the broad variation of published approaches, it is commonly accepted that highest possible sensitivity, robustness, and throughput are still the most urgent needs for the field. While many labs have focused on multiplexing to achieve these goals, label‐free SCP is a highly promising strategy as well whenever high dynamic range and unbiased accurate quantification are needed. We here focus on recent advances in label‐free single‐cell mass spectrometry workflows and try to guide our readers to choose the best method or combinations of methods for their specific applications. We further highlight which techniques are most propitious in the future and which applications but also limitations we foresee for the field.

**Abbreviations**
autoPOTS: automated preparation in one pot for trace samples
CTO: chronic total occlusion
CV: coefficient of variation
cv: column volumes
CyTOF: cytometry by time‐of‐flight
DDA: data dependent acquisition
DDM: *n*‐dodecyl β‐D‐maltoside
DIA: data independent acquisition
DTT: dithiothreitol
FAIMS: high‐field asymmetric‐waveform ion‐mobility spectrometry
FDR: false discovery rate
GV: germinal vesicle oocytes
HPLC: high pressure liquid chromatography
HRMS1‐DIA: high resolution mass spectrometry data independent acquisition
IAA: 2‐iodoacetamide
IPAD: integrated proteome analysis device
IVF: in vitro fertilization
IVM: in vitro matured
IVO: in vivo matured
LC: liquid chromatography
LF‐SCP: label‐free single cell proteomics
LIT: linear ion trap
mPOP: minimal proteomic sample preparation
MS: mass spectrometry
nanoPOTS: nanodroplet processing in one pot for trace samples
nanoSPLITS: nanodroplet splitting for linked‐multimodal Investigations of trace samples
nPOP: nano‐proteomic sample preparation
OAD: oil air droplet
µPAC: µ‐pillar array column
PASEF: parallel accumulation serial fragmentation
PCR: polymerase chain reaction
PIP: peptide identity propagation
pSCoPE: prioritized Single Cell ProtEomics
RT: retention time
SCoPE: single cell proteomics by mass spectrometry workflow
SCP: single cell proteomics
SPD: samples per day
TCEP: tris(2‐carboxyethyl)phosphine
TFE: 2,2,2‐trifluoroethanol
TMT: tandem mass tag
TOF: time of flight
UMAP: uniform manifold approximation and projection
WISH DIA: wide isolation window high‐resolution MS1‐DIA
WWA: wide window acquisition

## 1 INTRODUCTION

In contrast to the already well‐established single cell genomics and transcriptomics techniques, single cell proteomics (SCP) is still in its infancy. As a young and emerging field, SCP attracts tremendous attention because the proteome defines cellular identity and function. SCP therefore has enormous potential to not only understand cellular diversity but also in investigating pathogenesis. As such it is highly relevant in diagnostics and for the development of therapies for many diseases such as cancers.

**FIGURE 1:**
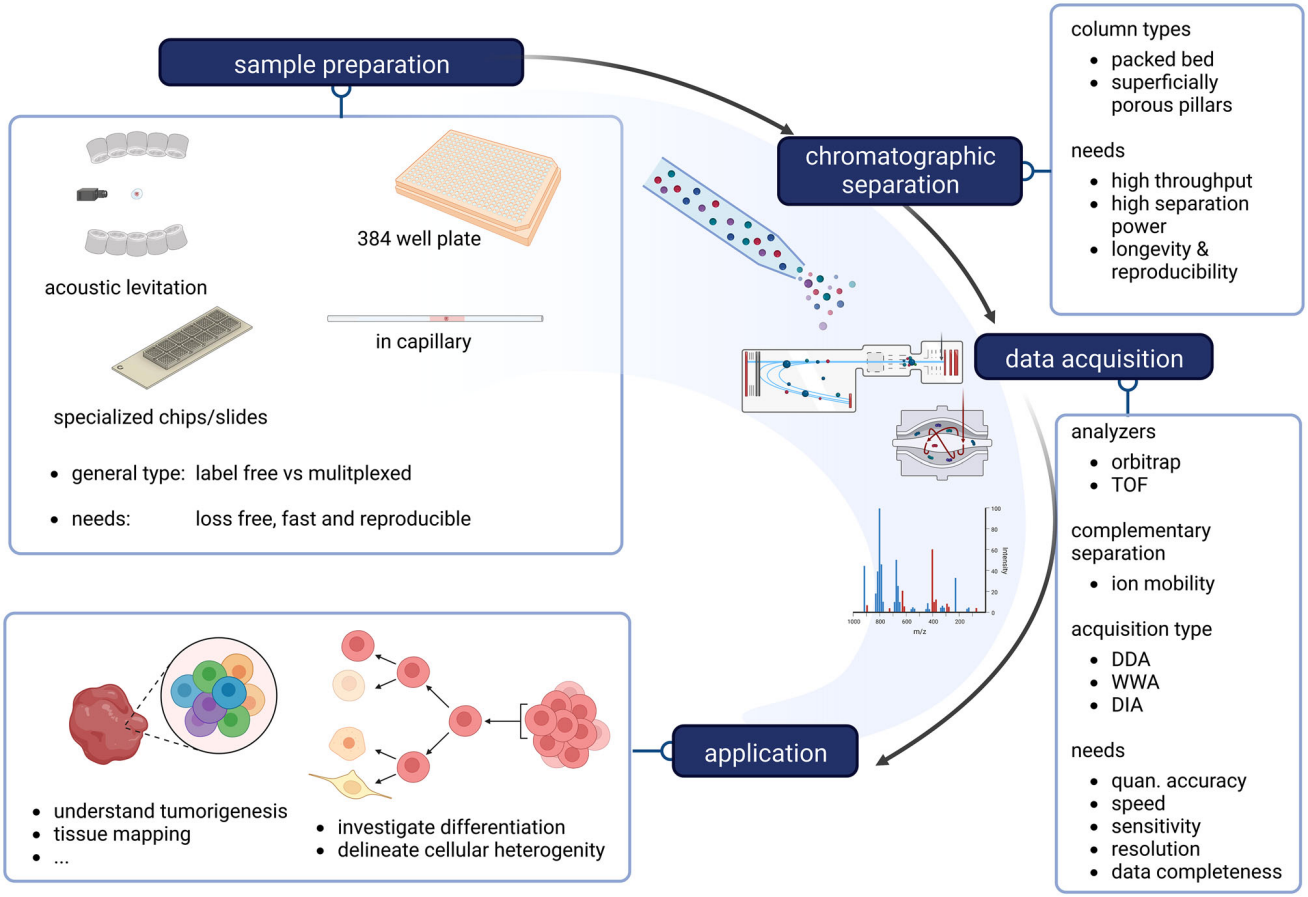
Typical steps of a label free single cell proteomics workflow and its potential applications.

In 2004, the first publication mentioning “Single Cell Proteomics” was listed on PubMed. Since then, the total number has increased exponentially to 227 by the end of 2022 with 70 of them published alone in 2022 highlighting the tremendous increase of interest in this field (Figure 1).

SCP, just like the other single cell omics fields, seeks to identify and characterize rare cell types or cellular (sub)populations in an unbiased way which would remain unnoticed in classical bulk analyses. As the proteomic contribution of rare cells within a bulk sample is limited, critical changes in these cells’ proteome may be missed but could potentially have substantial biological implications. Tumor heterogeneity, hematopoiesis, neurobiology, or cellular development are important examples where SCP could provide valuable new insigts [1, 2, 3, 4, 5].

Single cell sequencing would be unthinkable without nucleotide amplification. Polymerase chain reaction (PCR) catapulted nucleotide analysis into a new era, enabling technological breakthroughs in science and paving the way for society‐transforming applications such as COVID‐19 diagnostic tests. In proteomics and particularly in SCP a PCR for proteins would certainly be a gamechanger and lead to similar transformations in the field. Unfortunately, an innovation to amplify proteins is not in sight and it is quite doubtful whether it ever will be. Instead, the field tries to deal with the analysis of infinitesimal low quantities and relies on lossless sample preparation combined with highest sensitivity mass spectrometers.

Initially limited to very large cells such as blastomeres or oocytes [6, 7], SCP has matured substantially and now allows the identification and quantification of more than a thousand proteins for many cell types [8, 9, 10, 11, 12]. While the low abundance of protein sample material still poses a substantial challenge, major technical advancements in loss‐free sample preparation, nanoflow liquid chromatography (LC), and mass spectrometry (MS) as well as data analysis algorithms have advanced the field of SCP considerably. Initially, most SCP studies were utilizing a classical label‐free data dependent analysis (DDA) workflow. To achieve highest throughput and increase sensitivity, the field is now shifting towards sample multiplexing employing isobaric labeling such as tandem mass tag (TMT)‐labeling as well as non‐isobaric labeling [13, 14, 15]. By combining up to 18 cells using TMTpro into a single sample, sensitivity or rather protein abundance per analytical run is substantially improved by a factor of 18. Including a carrier proteome in TMT‐labeling workflows, one can further increase peptide signal intensities. To this end, one TMT channel, termed the carrier channel, is populated with up to 200 single cells, with the adjacent one or two channels being left out to avoid contamination spilling over from the carrier channel, while the remaining channels are populated by single cells. This results in triggering more and richer fragmentation spectra leading to a significant increase in peptide and protein identifications, while still allowing relative quantification based on the reporter ions of the respective TMT channel [16]. This method, originally termed single cell proteomics by mass spectrometry workflow (SCoPE)‐MS, has been introduced by Budnik et al. in 2018 [16] and the use of multiplexing and carrier proteomes in SCP has since been adopted widely [17, 18, 19].

Still, TMT‐labeling workflows suffer from several disadvantages. First, full utilization of all TMT18plex channels requires very high resolution MS2 spectra of about ≥50,000 at 150 *m*/*z* which limits the approach to Orbitrap based instruments. Long transient times of around 100 ms in the Orbitrap mass analyzer are required to reach the necessary resolution [20, 21]. Second, MS2‐based quantitation after TMT‐labeling is well‐known to suffer from ratio distortion due to unintended co‐isolation of multiple peptides [22, 23, 24]. While the extent of ratio distortion is larger the wider the isolation window is set, even at narrow isolation windows, ratio distortion from co‐isolation cannot be completely avoided. Thirdly, the dynamic range is very limited, and quantification might be negatively affected by highly abundant carrier channels. The majority of the peptide ions collected before fragmentation will arise from the carrier proteome whereas only few ions are collected from the actual single cells resulting in quantification inaccuracy [14, 25, 26]. Fourthly, data independent analysis (DIA) is becoming more and more popular in the field but is challenging to utilize in combination with isobaric labeling [27].

**FIGURE 2:**
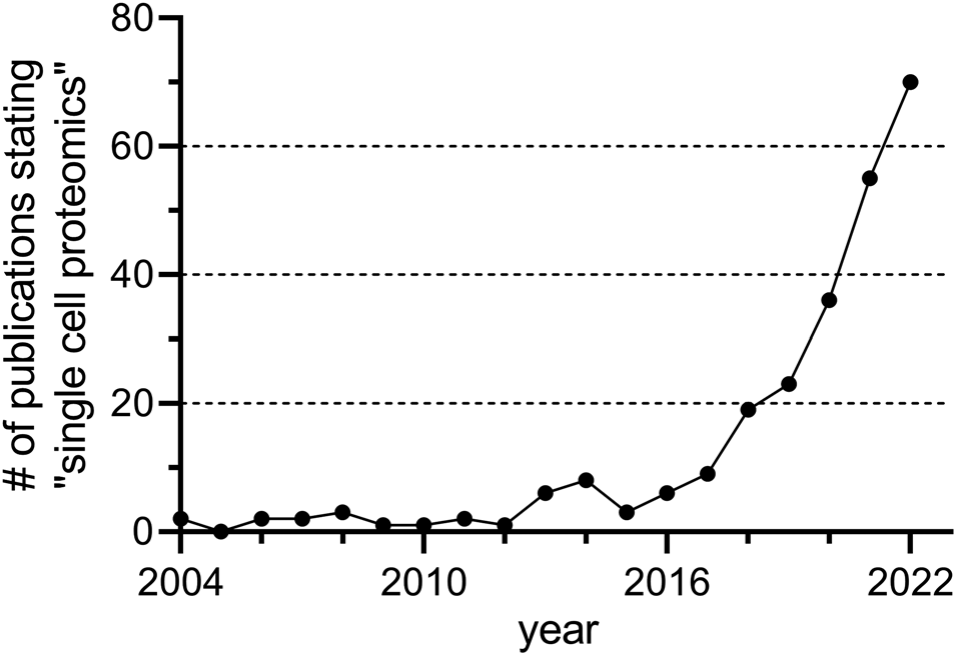
Number of publications bearing the term “Single Cell Proteomics” listed on PubMed for each year since first mentioned in 2004.

Hence, label‐free single cell proteomics (LF‐SCP) presents a viable and complementary option that does not introduce any bias, since no carrier is used. By this they outcompete multiplexed approaches thanks to higher quantitative accuracy. In this review, we focus on label‐free approaches to investigate single cells and discuss strengths and limitations, current challenges, and future perspectives for the field. Typical workflow steps and applications are summarized in Figure 2.

## 2 SAMPLE PREPARATION FOR LF‐SCP

Proper sample preparation is likely the most crucial step of single cell workflows as all further steps relies on its sensitivity, robustness, and reproducibility. Efforts made to process single cells or ultra‐low inputs label‐free are summarized in Table 1.

**TABLE 1:** Overview of parameters used in LF‐SCP sample preparation workflows.

Publication year	Protocol name	Processing volume	Container type	Cell isolation	Cell type	Lysis	Reduction and alkylation	Digestion	Cleanup	LC	MS	# Proteins identified from one cell		Comment
Specht et al. 2018 [40]	mPOP	1–1.5 µL	Well plate	FACS	HEK293, U937	Freeze‐heat cycle	No	Trypsin 4 h 37°C	None, direct injection	25 cm packed bed RP	Orbitrap; DDA	Not published	Not published	Applied also to multiplexed workflows
Leduc et al. 2022 [41]	nPOP	20–30 nL	Fluorocarbon coated slide	cellenONE	Jurkat, melanoma cells	100% DMSO	No	Trypsin @90 ng/µL 4 h	None, direct injection	25 cm packed bed RP	Orbitrap; DDA & DIA	Not published	Not published	
Shao et al. 2018 [42]	iPAD	2 nL	In capillary	Manual, microscope	HeLa	Simultaneously, 30 min, 50°C +sonication, no reduction, and alkylation			None, direct injection	In‐house made packed bed RP	Orbitrap; DDA	181	328	
Li et al. 2018 [30]	OAD	100 nL	Specialized chip	Semi‐automated, microscope	HeLa	Rapigest & sonication	TCEP + IAA	4 h LysC + 16 h trypsin at 37°C; under oil	None, direct injection	In‐house made, 15 cm packed bed RP	Orbitrap; DDA	36	–	
Matzinger et al. 2022 [11 ]	–	1 µL	384 well plate	cellenONE	HeLa, K562	simultaneously in mix of DDM and trypsin, 2 h at 50°C, no reduction and alkylation			C18 trap column	5.5 cm superficially porous pillars	Orbitrap; DDA, WWA & DIA	>1000	Up to 1791	
Johnson et al. 2022 [43]	–	0.5 µL drop	In capillary	Manual, microscope	HeLa	75% Formic acid, 5 psi, 27 s	No	No	None, direct injection	Capillary electrophoresis	Orbitrap; DDA	26±10	48	Top down
Matsumoto et al. 2022 [34]	–	1–4 µL	Container‐less	Flow cytometer	HEK293	Hypotonic lysis in ddH_2_O	No	Trypsin, 30–90 min, 25–50°C	C18 trap column	In‐house made C18	No details given	–	700/50 cells	50 cells
Mund et al. 2021 [44]	Deep visual proteomics	1 µL	384 Well plate	Laser microdissection	FFPE tissue, U2OS	20 mM ABC, 95°C for 30–60 min	No	4 ng LysC , 4 h at 37°C + 6 ng trypsin, 16 h at 37°C	none, direct injection	50 cm in‐house made packed bed C18	TOF; DDA & DIA	–	–	>80 cells
Brunner et al. 2022 [39]	–	1 µL	384 Well plate	FACS	HeLa	72°C in 20% acetonitrile 30 min + sonication	No	37°C 1 ng trypsin/LysC mix 16 h	Evo Sep‐StageTips	15 cm packed bed C18	TOF; DDA & DIA	∼500	843 (DDA), 2083 (DIA)	
Zhu et al. 2018 [37]	nanoPOTS	200 nL	Chip nanowells	FACS	HeLa	DDM and DTT 70°C 1 h	IAA	0.25 ng Lys‐C, 4 h +0.25 ng trypsin, 6 h	SPE column	50 cm packed bed RP	Orbitrap; DDA	211	669	
Liang et al. 2021 [29]	autoPOTS	4 µL	384 well microplate	FACS	HeLa, lymphocytes	DDM, sonification	DTT, IAA	1 ng LysC 3 h 37°C + 1 ng trypsin 12 h 37°C	SPE column	45 cm in‐house made packed bed RP	Orbitrap, DDA	301	–	Fully automated
Cong et al. 2021 [38]	nanoPOTS	200 nL	chip nanowells	In‐house built robot	HeLa, neurons	DDM and DTT 70°C 1 h	IAA	0.25 ng Lys‐C, 4 h +0.25 ng trypsin, 6 h	SPE column	Ultra‐narrow‐bore 50 cm packed bed	Orbitrap + Iontrap, DDA	1085	–	
Petrosius et al. 2022 [45]	–	1 µL	384 well	FACS	HEK	TFE, 95°C 5 min	No	2 ng trypsin + benzonase, 16 h 37°C	Non‐porous 5 µm C8 pillar trap column	50 cm superficially porous pillars	Orbitrap, WISH‐DIA	1666	1972	Matching to gas‐phase‐fractionated library

Preparation of ultra‐low input samples is limited by their extremely low peptide concentrations. Peptide losses by adsorption to surfaces are among the biggest concerns. In addition, high excess rates of protease over protein have to be used since protease activity is also concentration dependent [28]. This, however, leads to intense background signals of the used protease resulting in signal suppression that limits reachable dynamic range. Poor reproducibility is another important key limitation of SCP workflows. Random errors, differences in sample transfer steps or storage, that would not be of concern when analyzing higher‐concentration bulk samples yield in significant changes in results of SCP workflows.

To alleviate these challenges, researchers minimize the volumes used during sample preparation. This lowers the covered surface area available for adsorption processes and increases peptide and protease concentration at the same time. Most protocols therefore process their samples in volumes ranging from few microliters down to nanoliters (Table 1). As a tradeoff, this introduces reproducibility issues due to sample drying and inaccurate pipetting of low volumes. Both can be largely avoided by workflow automatization including specific measures to reduce sample drying such as working under high humidity and artificial hydration [29] or working under a protective oil layer [12, 30]. The reader is referred to another excellent review for a comprehensive overview on the degree of automatization in current workflows [31].

Another crucial point to reduce sample losses is to omit sample transfer or cleanup steps whenever possible. Even for protein handling amounts of 50 and 2 µg sample loss ranges from 15% to 89% respectively upon multiple transfers [32]. A single HeLa cell is estimated to contain much less material, only 150–250 pg protein [33], which highlights how critical it is for the success of any single cell experiment to avoid sample loss. As a result, most ultra‐low input workflows complete all processing steps within a single pot or even container‐less [34].

Cleanup steps are required for most bulk proteomic workflows to remove MS incompatible reagents as urea, SDS, or cellular debris. In contrast, especially label‐free workflows with only a single cell as input usually cannot afford to risk sample losses or to introduce sample variability through a cleanup step. This leads to the usage of MS compatible detergents, such as *n*‐dodecyl β‐D‐maltoside (DDM) [35, 36] or skipping of otherwise common steps such as reduction and alkylation (Table 1). The low amount of sample here gives the advantage of less cell debris or nucleotides present, eliminating the need for their removal before LC‐MS analysis. However, some workflows successfully apply online sample cleanup and concentration using loading columns [29, 34, 37, 38] or StageTips [39].

## 3 LIQUID CHROMATOGRAPHIC SEPARATION

**FIGURE 3:**
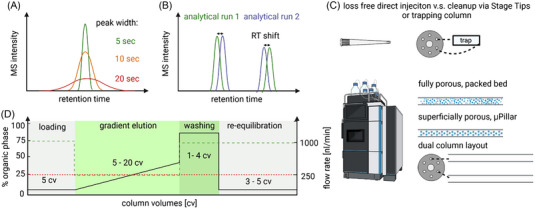
Details of chromatography setting influence sensitivity, reproducibility, and throughput. (A) The same peptide in equal quantity produces higher intense sharp and lower intense broad peaks. (B) Chromatographic reproducibility is essential for retention time (RT) prediction as well as match between run (MBR) (C) RT, peak width, and reproducibility are highly influenced by the used column type and length. Dual column settings can speedup resulting run‐to‐run time. (D) Typical high pressure liquid chromatography (HPLC) gradient start at 1%–2% organic buffer and range to 35%–45% organic buffer. Over 5–20 column volumes (cv). Typical flowrates in proteomics are in the nanoliter range (100–300 nL/min, red dashed line). Throughput can further be boosted by fast loading and equilibration (green dashed line).

The tremendous importance of chromatographic separation performance for (complex) proteomic samples was already demonstrated back in 2016 by Shishkova et al. [46] Indeed, nanoflow liquid chromatography is most commonly used in proteomics since peptide ionization by electrospray ionization is improved at low flow rates, resulting in improved sensitivity[47, 48]. While long gradients at low flow rates show best separation power, their bottleneck is speed. In case of very long gradients, peak‐broadening again occurs causing less intense peaks. This limits sensitivity, which is crucial for ultra‐low inputs (Figure 3A). To keep pace with multiplexed approaches, shortest possible run‐to‐run times are pursued in LF‐SCP. In conclusion, shortest possible gradients employing low flow rates would be ideal to achieve the best sensitivity and throughput. In line with these considerations, there indeed seems to be a sweet spot for gradient length. Furthermore, scan speed and required fill times of the MS are also factors that need to be considered for the choice of the minimal gradient length. Too long gradients on the other hand suffer from peak broadening and loss of sensitivity. Our data show, that for a single cell level input of 250 pg using a DDA method, 808 protein groups are identified with a 10 min active gradient, which is increased to 1485 protein groups when using a 30 min gradient and drops again down to 1320 protein groups for a 50 min active gradient [49].

This again highlights that sharp chromatographic peaks are desirable. They were further shown to be triggered closer to their apex in data dependent acquisition (DDA) thanks to lowered median offset time between actual peak apex and MS2 spectra triggering time [50]. Highly resolved peaks additionally reduce the chance for co‐isolation of precursors within their isolation window [51] (if not intended by broad *m*/*z* windows, see description of “WWA” in section “Ultrafast mass spectrometry for label‐free (LF) SCP”). While this is less important for label‐free approaches, minimizing co‐isolation is essential for achieving high accuracy for reporter ion quantification in multiplexed workflows. A reduced overlap across (sharp) peaks further leads to lowered competition between peptides in the ion source which augments their intensity in the resulting MS spectrum.

Flow rates as low as 790 pL/min using 2 µm inner diameter open tubular columns have enabled identification of more than 100 and ∼1000 proteins from sub‐single‐cell amounts of 0.75 or 75pg respectively in a 30 min active gradient [52]. Although these results are impressive, such low flow‐rates could only be achieved by splitting the solvent flow, and further improvements will be needed to allow for a loss‐free sample injection of a single cell [50]. As a trade‐off, low flow rates require long time periods for column loading, washing, and re‐equilibration. This has a negative effect on throughput, since the actual run‐to‐run time is much longer than the active gradient.

A large‐scale plasma study demonstrated that indeed the chromatographic system is responsible for 80% of MS idle times. Loading, washing, and equilibration may take up to 20 min or more which makes short gradients for high throughput less attractive [53, 54]. Increasing flowrates before and after the active gradient largely addresses this issue (Figure 3D). With this, run‐to‐run times were shown to be successfully shortened to 20 min for a 10 min gradient [45, 49] or 7.4 min for 5 min active gradient [11] allowing the measurement of 72 and 194 samples per day (SPD), respectively. Of note, modern high pressure liquid chromatography (HPLC) systems like the Vanquish Neo (Thermo Scientific) enable an automated speedup of loading and equilibration by setting a maximum flow rate and/or pressure as limit.

In a recent preprint from our lab, we evaluated the use of ultrashort gradients, down to 5 min with a 7.4 min final run‐to‐run time for single cell inputs. Here, we are limited by the Orbitrap scan speed that leads to loss of proteomic depth when using such short gradients. MS1‐only data acquisition is an exciting yet immature alternative that enabled identification of 750 proteins from single cell input in 5 min [55]. In addition, quantitative accuracy was reported to be superior over standard DDA for the MS1‐only approach [56].

Another chromatographic method designated for high throughput SCP is Whisper implemented by Evosep [57]. Using low flowrates of only 100 nL/min and short gradients, 20–40 SPD can be processed and this was already successfully used to quantify up to 2000 proteins from a single cell [39].

The usage of multiple columns (Figure 3C) is another smart strategy to improve throughput. While one analytical column is used for peptide separation the other is washed and re‐equilibrated to keep MS downtime low. Such a setting was already successfully used to analyze 200 single cell proteomes a day using a label‐free approach [58].

Such tricks should however be considered with care to maintain retention time (RT) stability across runs. Carefully adopted systems allow to maintain RT variation between both columns below 2% [59]. However, especially for very short gradients this might already be problematic, even more in case of flipped instead of linear shifts of RTs (Figure 3B) for match between run (MBR) algorithms or for software tools relying on RT prediction to score peptides. Arguably an excellent alignment across replicates is also of greatest importance to reduce missing values in biological studies with large sample numbers as is for targeted approaches with scheduled triggering of precursors of interest.

Trap‐and‐elute setups are commonly used to deliver samples onto the column. Thereby the analyte is first loaded to a trapping column between two ports of a switching valve at high flow rate. After that, the analyte is either eluted into the analytical column via the gradient or by inversing the flow direction on the trapping column (backflushing) (Figure 3C) [60]. Their clear advantage is a shortened loading time. In addition, they allow to wash away solvents or other impurities which can be considered beneficial for single cell digests where usually no offline cleanup is done. Alternatively customized StageTips can be used as disposable trapping column. This system was implemented by the Matthias Mann and coworkers in collaboration with Evosep [57] and shows excellent compatibility with current LF‐SCP workflows [39, 61]. The disadvantage of trapping columns and StageTips is that sample retention can be incomplete, leading to sample losses. SCP injection volumes are usually small enough to be applied directly onto the analytical column in a reasonably short time. In conclusion, there is no consensus whether or not trap and elute setups should be used in SCP workflows (see Table 1).

Analytical columns with the highest possible chromatographic resolution are used to separate peptides in SCP workflows. Packed bed type columns are commonly used. Increasing column length and decreasing silica particle diameter has historically led to improved performance and increased operating pressures [61, 62, 63]. Two decades ago, the introduction of highly ordered porous pillar arrays revolutionized the field due to their superior chromatographic resolution while backpressure is significantly reduced, allowing for higher flow rates and throughputs [64, 65]. The highly ordered layout reduces dispersion due to flow path variability which is a significant cause of peak broadening in conventional columns [66]. Subsequently superficially porous and non‐porous materials were introduced. Although the superficially porous particle architecture has been known for more than 50 years [67], its application in µ‐pillar array columns (µPACs) for low and ultra‐low input samples was demonstrated very recently [44, 68]. µPAC columns bearing nonporous pillars were further developed for ultra‐low input amounts and showed superior performance compared to conventional porous particle packed columns [69].

## 4 ULTRAFAST MASS SPECTROMETRY FOR LF‐SCP

Based on the requirements of ultra‐low‐input samples and compatible optimal chromatographic settings (see section “liquid chromatographic separation” above), mass spectrometers need to support fastest possible scan speeds at highest possible sensitivity. Orbitraps and time of flight (TOF) detectors are the main players in the field. Whereas Orbitraps have highest resolving power and are very sensitive allowing for a limit of detection in the low attomolar range, they are limited in speed with the most advanced generation reaching speeds of 45 Hz (Thermo Orbitrap Ascend Tribrid). In contrast, TOFs are limited in resolution but outplay their competitors in speed reaching scan rates of more than 120 Hz (timsTOF Pro2, Bruker) or even up to 133 Hz (ZenoTOF, SCIEX). While a sufficient resolution of min > 50,000 at 150 *m*/*z* is crucial to resolve the 6 mDa reporter ion mass difference in highly multiplexed samples [20, 71] and cannot be resolved using state of the art TOF devices, this is of less importance for label‐free workflows. Speed therefore seems to be the greater advantage here but still the sensitivity of TOFs was a hurdle in early single cell approaches. Recently, the Mann and coworkers [39] modified the geometry, glass capillary, and ion optics of a their timsTOF Pro instrument to improve its robustness against contaminations and to increase ion transmission by a factor of 4.7. With this the updated device, now called timsTOF SCP, is sensitive enough to perform single cell level studies. Using the timsTOF SCP and benefiting from the high TOF scan speed, they demonstrated the successful and reproducible quantification of >3900 proteins from 1 ng of HeLa input. Using a data independent acquisition (DIA, see also below), the resolution coefficient of variation (CV) was <10% and data completeness was at 92 % across five replicates.

In contrast, the lowered scan speed of Orbitraps results in less datapoints per peak triggered and higher CVs especially for DIA methods with sometimes long duty cycles. For DIA, quantification on MS2 level is preferred as it is believed to be more accurate by better dealing with co‐elution bias [72, 73]. There are however tricks to reduce the cycle time to eventually end up with more datapoints per peak for quantification. Using high resolution mass spectrometry data independent acquisition (HRMS1‐DIA) [74] the *m*/*z* range of interest is segmented and intermediate MS1 scans are scheduled thereby increasing the number of available MS1 scans at cost of MS/MS scans. This potentially improves quantification on MS1 level but hampers it on MS2 level. In a recent preprint [45] from Erwin Schoofs group this strategy was shown to not only yield in more identifications but also and more importantly yielded in more datapoints per peak, hence lowering CV and improved quantitative precision. Another adoption of this trick termed wide isolation window high‐resolution MS1‐DIA (WISH‐DIA) combines HRMS1‐DIA with isolation windows widened to 10–100 *m*/*z*. WISH‐DIA improves protein identifications most ideally at a 40 *m/z* isolation window, and allows for longer injection times and higher resolution without affecting total cycle time.

Another alternative to Orbitraps and TOF analyzers are linear ion traps (LITs). Although rarely used, their benefit for low input samples has been recently demonstrated [75]. Similar to TOFs, they allow for very fast scanning rates of up to 125,000 Da/s [75] making them especially exciting for DIA methods and for ultrafast chromatographic separations. It was again the lab of Erwin Schoof presenting an optimized DIA method using an Orbitrap for MS1 and an LIT for MS2, that clearly outperforms measurements using the Orbitrap for sample inputs <10 ng both by means of peptide identifications and robust quantification. It seems that in addition to speed, the main advantage of LITs is sensitivity, which is superior over Orbitrap sensitivity. However, this comes with a high noise level as trade off and results in difficulties in data analysis. While highly multiplexed (i.e., TMTPro) samples cannot be resolved using the LIT due to its limited resolutions, the signal to noise ratio can be significantly improved when combining with a FAIMSPro interface to successfully quantify representative proteomes from ultra‐low inputs in label‐free approaches with high throughput [61].

Besides mass detectors, ion mobility as additional separation dimension can decrease chemical background noise to enhance sensitivity. Compatible with Orbitrap instruments, the FAIMSPro prevents neutral ions from entering the mass spectrometer, improving robustness and sensitivity for proteomics experiments [76, 77]. The resulting increase in signal over noise was shown to improve proteome coverage especially for low input samples. Furthermore, high‐field asymmetric‐waveform ion‐mobility spectrometry (FAIMS) improves quantitative precision both using data dependent and independent acquisition [78, 79]. As an alternative to FAIMS, the timsTOF devices from Bruker introduce a so‐called trapped ion mobility funnel that adds the collisional cross section of analytes as additional dimension to LC‐MS enabling improved proteome coverage at reduced analysis time [80, 81]. Similar to FAIMS, the signal to noise ratio can be increased by excluding singly charged ions from analysis. These devices have recently been used very successfully for low inputs down to individual cells. Brunner et al [39]. identified more than 2000 proteins from one cell (see also Table 1) and in a recent technote of Bruker [82] more than 1500 protein groups were quantified using a label‐free workflow within the proteoCHIP.

The choice of proper data acquisition parameters is at least as influential as the choice of the instrumentation. As highlighted before, pushing sensitivity and throughput is amongst the most important aspects for ultra‐low input samples. This results in methods with low target intensities and high injection times to enable deeper proteome coverage. Currently DDA is arguably the most prominent way to go in proteomics. Here an MS1 full scan is acquired, and top n precursor ions are selected based on pre‐defined parameters for fragmentation. This is repeatedly done throughout the entire analytical gradient and enables a straightforward identification and quantification of peptides. However, it adds a high degree of stochasticity to the data which might lead to lowered run‐to‐run reproducibility especially for single cell level inputs where low abundant precursors might be (randomly) selected or not. In DIA, the entire mass range of interest is divided into bins of either fixed or variable sizes usually ranging from 10 to 30 *m*/*z*. As already aforementioned, even wider windows up to 100 *m*/*z* have been tested and wider than usual bins were shown to be beneficial for SCP [45]. In theory all precursor ions in the entire mass range are fragmented and are scanned sequentially. This promised to facilitate improved proteome coverage and reproducibility across runs but due to increased spectrum complexity, data analysis is more challenging [83]. In recent years, DIA experiences a renaissance as the advantages are predominant and data analysis is powerful enough to handle complex chimeric spectra nowadays (see Section 5). Of note, recently two‐way communication‐based strategies gained popularity. Advanced algorithms are thereby leading a complex decision process on collection time, isolation window or fragmentation energy on‐the‐fly. This can also be used to enable a close matching in used time windows for monitoring specific peptides in targeted approaches. This however requires real time searching and spectral matching. Thanks to more and more powerful yet affordable computers and advanced software, such approaches will likely dominate the field in the near future [84, 85, 86].

In line with the trend of more complex spectra, our lab also pioneered in testing ultra‐wide windows in DDA (termed wide window acquisition [WWA]) workflows to on purpose fragment >1 precursors. Using WWA the number of peptides identified form an individual spectrum was boosted to up to 10, which improves the overall proteome coverage without the need to elongate gradient time. By using WWA more than 1000 proteins were identified from single cell input compared to max 700 using a conventional isolation window of 1 Thompson [69].

Another simple but innovative and clever strategy to improve coverage, speed and at the same time remove stochasticity, is to record data without any MS/MS fragmentation. Here, identifications are based exclusively on high resolution precursor masses and RT prediction. Skipping fragmentation and recording of the resulting MS2 spectra frees up measurement time by factor 10–20 and allows for proteome‐wide analyses within 5 min. Its quantification efficiency was recently demonstrated to be comparable to multiplexed TMT‐based approaches and to DIA workflows [55, 56, 87, 88]. The enormously short gradients are of high potential for label‐free proteomics as they allow to reach throughputs of roughly 200 SPD. In our lab, we successfully tested MS/MS free data acquisition to identify close to 750 proteins from a single cell level input [11]. This approach remains to be evaluated by more labs to gain confidence in reliability of identifications not based on fragment spectra, but the authors of this review believe it has the potential to move the single cell field forward a lot in future.

**FIGURE 4:**
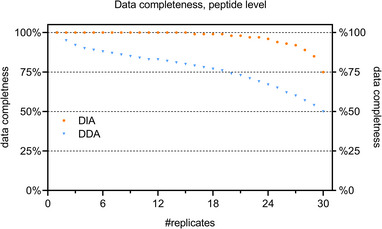
Degree of data completeness (1/fraction of missing values) is dependent on data acquisition. 250 pg of tryptic HeLa digest were repeatedly injected from the same vial, creating a dataset without any biological variability. The fraction of peptides found in n replicates is plotted when either using data dependent analysis (DDA) or data independent analysis (DIA) to acquire data. All data was recorded on the same Orbitrap Exploris 480 (Thermo) using the same 5.5 cm µ‐pillar array columns (µPAC) analytical column and a 20 min active gradient. Raw data can be accessed free of charge via the ProteomeXchange Consortium in the PRIDE [90] partner repository with the dataset identifier PXD039208. For DDA data was analyzed using CHIMERYS and quantified with apQuant [91] with match between run (MBR) enabled. For DIA Spectronaut v16 was used in directDIA mode including spectral matching across all files.

Among all proteomic studies, missing values are an important issue when it comes to quantitative comparison of replicates for large sample cohorts. As single cell studies ideally require hundreds to thousands of individual cell samples to investigate cellular heterogeneity, this problem becomes severe especially for label‐free single cell workflows. Imputation of missing values may alleviate this issue. However, its universal validity is controversial and the correct imputation method must be wisely chosen. The reader is referred to another excellent review on that topic [89]. In conclusion, there is an urgent need to lower missing values by the choice of a proper data acquisition strategy. In line with expectations, our data shows that DIA clearly outperforms DDA in terms of data completeness (Figure 4) again highlighting that DIA might dominate the field soon.

Another strategy, prioritized Single Cell ProtEomics (pSCoPE), seeks to improve reproducibility across runs by exclusively focusing on specific peptides of interest. By prioritizing peptides already identified, data completeness and sensitivity can be boosted in subsequent runs. Using pSCoPE, Huffman et al. increased final proteome coverage twofold [86].

## 5 DATA ANALYSIS PLATFORMS FOR LF SCP

MS2‐based peptide identification algorithms have improved tremendously over the last 20 years, resulting in numerous excellent software solutions from both, commercial and academic developers (e.g., Sequest HT [91], Spectronaut [92], DIA‐NN [71], MaxQuant [93, 94], PEAKS Studio [95], and MSFragger [96]). However, all these algorithms have been developed and optimized for proteomic bulk samples with a number of assumptions including, (i) that the number of fragment ion peaks matches between theoretical and observed spectrum, (ii) fragment ion peak intensities are substantially greater than background signals, and (iii) repeated spectra of the same precursor will look near identical. A recent study has demonstrated that these assumptions might not be fully correct for single cell spectra, for which Boekweg et al. have found loss of annotated fragment ions, blurring between signal and background noise based on reduced ion intensity as well as distinct fragmentation patterns as compared to bulk spectra [97].

Compared to typical bulk proteomics samples, the greatest challenge for SCP is that considerably fewer proteins can be identified and quantified. While around 6000 – proteins can be identified from HeLa whole cell lysates with relative ease, most single cell studies fail to identify more than 1000 proteins from a single cell as shown in Table 1. Unsurprisingly, even sensitive instruments such as the Orbitrap require long ion accumulation times of >100 ms to collect sufficient ions for interpretable fragmentation spectra, which negatively impacts cycle time prolonging duty cycle times and hence deteriorating the number of datapoints per peak & consequently quantification. While this is not such a massive limitation in DIA methods, in DDA aiming to fragment only a single precursor at any given time it critically reduces the number of precursors that can be triggered. The AI‐driven search algorithm CHIMERYS in contrast allows to identify >10 peptide sequences from a single MS2 spectrum thereby increasing the number of identified peptides and proteins per sample typically by 100% and 50%, respectively, when using WWA applying 4–12 *m*/*z* precursor isolation widths [69]. CHIMERYS does this by predicting peptide properties such as RT and peptide fragment intensities using the INFERYS 2.0 deep learning framework, that was trained on millions of spectra from tryptic and non‐tryptic peptides [99]. This has allowed us and others to achieve proteomic depths in single cell samples of over 1000 proteins at an false discovery rate (FDR) of 1% without the use of libraries or identification transfer between samples [11].

As described in the previous chapter, missing values are a substantial challenge especially when using traditional DDA‐based approaches. Already at the bulk level for small DDA studies with only 18 samples, the typical fraction of missing peptide values is ≥17%, which becomes substantially worse in single cell studies (see Figure 4 and recent literature [100, 101]). MaxQuant, as one of the most popular data analysis tools, manages to reduce missing values by peptide identity propagation (PIP) via accurate mass and RT mapping on the MS1 level using the match‐between‐runs (MBR) algorithm [94, 102]. Recently, however, more sophisticated approaches to reduce missing values have been published including the IonStar[103] and the IceR[100] algorithm. IonStar avoids the need to detect isotope peak patterns completely, which would limit sensitivity. Instead, it solely uses ion‐based PIP applying direct ion current extraction instead[103]. In contrast, IceR combines the use of feature‐based and ion‐based PIP in a hybrid strategy, reaching very high degrees of sensitivity and data completeness. This reduces the fraction of missing values drastically to a few percent in most cases, whereas missing values for traditional tools such as MaxQuant even using MBR are in the range of >15% for bulk analyses and >50% for single cell studies [100].

Another option to improve data completeness is the use of DIA DDA's inherent stochasticity (Figure 4). DIA typically reduces missing values substantially to less than 10% when 20 samples are analyzed and is therefore becoming increasingly popular both in regular bulk proteomics as well as low input and single cell studies [61, 104, 105]. Data analysis for DIA runs is typically performed with either Spectronaut [93] or DIA‐NN [72]. As the two leaders in the field of DIA data analysis, both of deliver excellent sensitivity and data completeness. While Spectronaut is available as stand‐alone software tool, DIA‐NN can be used either as a stand‐alone version or integrated into the FragPipe platform [106].

With the availability of the additional ion mobility dimension on timsTOF instruments, the classical DIA approach has been developed further to better utilize the ion current for improved sensitivity resulting in the diaPASEF [107] and the slice‐parallel accumulation serial fragmentation (PASEF) [108] methods. Both, diaPASEF and Slice‐PASEF make use of the correlation of precursor masses and their ion mobilities by synchronizing the quadrupole scanning window with the ion mobility elution thereby boosting throughput up to fourfold. This leads to up to fourfold more ions in the detector and improves sensitivity drastically. While diaPASEF uses a few (usually 2–5) of predefined, non‐overlapping *m*/*z* windows during ion mobility elution, Slice‐PASEF uses a more continuous approach. Thereby a high number of *m*/*z* windows (10–15) are used independent from each other in the *m*/*z* dimension, but each window is corresponding to a narrow ion mobility range (1/*K*0 = 0.03–0.045). Compared to diaPASEF, Slice‐PASEF demonstrated even improved sensitivity and quantitative performance for low input amounts of 10 ng but to understand its performance for LF SCP studies, analysis of lower inputs and real single cell samples is necessary.

## 6 APPLICATIONS OF LF‐SCP AND RECENT STUDIES

Initial research efforts in single cell method development have mostly aimed towards pushing technical boundaries to enable deeper proteomic coverage, improved data completeness and quantification to lay the foundation for relevant and reproducible biological and clinical studies. Indeed, these efforts are bearing fruit and label‐free proteomics has now matured enough to allow exploring more applied studies to investigate biological questions of high importance to society such as cellular development from stem cells to highly differentiated and specialized cells with major implications in tissue regeneration and in vitro fertilization (IVF) as well as many other fields. In oncogenesis, SCP could help researchers track the formation of drug resistance and highlight potential ways to circumvent it. SCP could also enhance understanding of the onset of various types of cancer to not just help treat cancer but even prevent it in the first place. Particularly for larger and medium sized cells such as oocytes, skin cells or hepatocytes LF‐SCP should be highly relevant, while very small cells such as lymphocytes or erythrocytes might dictate the use of multiplexing workflows due to the ultralow amount of initial protein available. Several recent manuscripts have applied LF‐SCP to biological questions and report encouraging results with first interesting insights into biology (Woo et al. [9]).

One of these works describes the heterogeneity on the single cell proteome level of human oocytes upon in vitro and in vivo maturation [109]. This is of particular relevance for IVF approaches. Guo et al [109]. sampled 36 human oocytes from 13 different donors and identified 2382 proteins of which 2094 could be quantified. The oocytes belonged to one of three conditions including immature germinal vesicle oocytes (GV) as well as in vitro matured *(IVM)* and in vivo matured (IVO) oocytes. A total of 176 proteins were found to be differentially abundant between GV and IVO oocytes and 45 between IVM and IVO oocytes. Among these proteins, maternal effector proteins were identified potentially related to the observed decreased fertilization, implantation, and birth rates using IVM oocytes. Next to the single cell proteome, also the single cell transcriptome was evaluated, which showed low correlation to the SCP data with correlation coefficients of <0.2 when comparing fold changes. This again highlights the added value of SCP data over single cell transcriptomics data. Interestingly, IVM oocytes displayed higher proteomic inter‐cell variability as compared to GV and IVO oocytes potentially suggesting homogenous oocyte states in vivo. This could also suggest a higher variability of IVM oocyte quality as compared to IVO oocytes, which would be consistent with the observed reduced biochemical and clinical pregnancy, as well as live birth rates when using IVM oocytes during IVF [110]. The authors investigated the potential origin of this heterogeneity and found significant correlation of the Estradiol/follicle ratios (E2/fol) with the Euclidean distance from each oocyte to the median of the group. This suggests that E2/fol level fluctuations may contribute to the heterogeneity of in vitro–matured oocytes. It has also been shown previously that low levels of E2/fol are associated with low implantation rates as well as higher risks of single and triple pronucleus formation and abortion [111].

In another study of interest, Li, et al. used mass‐adaptive coating‐assisted single‐cell proteomics (Mad‐CASP) methodology to investigate CD34^+^ peripheral blood mononuclear cells (PBMCs) in the arterial blood of chronic total occlusion (CTO) patients for comparison to healthy donors [112]. CTO is characterized by a (near) complete blockage of one or more coronary arteries for 3 months or longer leading to restricted blood flow to the heart and serious health complications such as a heart attack. Circulating CD34^+^ progenitor cells play important roles in vascular repair thereby affecting cardiovascular health and longevity, which renders them highly interesting targets for CTO research [113]. The authors’ Mad‐CASP approach includes the coating of the sample containers with a synthetic peptide to minimize adhesive sample losses. The synthetic peptide contains mostly hydrophobic amino acids with a tryptic cleavage site every five amino acids resulting in the liberation of short tryptic peptides upon digestion. This allows to also saturate LC‐surfaces such as tubings and columns thereby preventing sample peptide losses during chromatographic separation. The short length of the tryptic peptides leads to them not being detectable in the classical *m*/*z* range of tryptic sample peptides of 350+ *m*/*z* avoiding dynamic range issues in the MS. This system allowed the authors to identify 23%–63% more proteins when compared to uncoated vials. For the analysis of the CD34^+^ blood cells, the authors first generated 2000‐cell libraries for each of the six donors (three healthy, three CTO patients) from which they could identify 1592–2773 proteins for CTO patient cells as well as 3376–4195 proteins from healthy control cells. Harnessing these libraries, Li et al. could identify on average 844–870 and 931–1058 proteins for CTO patients and healthy donors, respectively, using MaxQuant and MBR. Separate clustering of healthy donor and CTO patient‐derived cells by uniform manifold approximation and projection (UMAP) analysis allowed the identification of three subpopulations for both. One subpopulation in both conditions was associated with artery occlusion, atherosclerosis, coronary artery disorder, and other CTO‐related occlusions, while another CTO‐derived subpopulation uniquely displayed proteins involved in vascular lesions. Another subpopulation found in both conditions expressed proteins involved in angiogenesis and vasculogenesis, while the third subpopulation in healthy was characterized by proteins with functions in arterial development. It therefore seems that CTO patients specifically feature a CD34^+^ cell subpopulation that could potentially act as a driver of the disease. Therapeutic intervention to reprogram this particular subpopulation could potentially allow to better manage CTO and help to improve CTO patients’ lives.

Even though the coverage obtained from LF‐SCP has substantially improved to provide fascinating results, additional depth would enhance its transformative power toward answering important biological questions well into the future.

## 7 CONCLUSIONS, PERSPECTIVES, AND FUTURE CHALLENGES

SCP, and particularly LF‐SCP, has matured is becoming widely available now to an increasing number of proteomics labs that had previously shied away from the technical obstacles. The ease of implementation of LF‐SCP allows scientists to enter the single cell field in a straightforward manner. Early studies of LF‐SCP in human fertility research and cardiovascular disease have resulted in relevant and encouraging data and should foreshadow the widespread application of LF‐SCP to many other burning biological and pathological questions in areas such as tumor heterogeneity, hematopoiesis, neurobiology, or cellular differentiation and organ development. LF‐SCP is expected to be most relevant to the investigation of larger cells with diameters >15 µm such as fibroblasts, macrophages, cardiomyocytes, or oocytes, while smaller cells would highly benefit from multiplexed approaches. However, LF‐SCP offers a high degree of quantitative accuracy and data completeness when utilizing DIA, which is not always the case for multiplexed approaches. In comparison to cytometry by time‐of‐flight (CyTOF), LF‐SCP offers a more comprehensive view of the cellular proteome representing a hypothesis‐generating approach, while the targeted approach of CyTOF rather facilitates hypothesis‐driven research by monitoring dozens of pre‐defined proteins rather than hundreds.

Diligent planning and avoidance of sample losses during the entire workflow starting with loss‐less sample preparation, powerful and rapid peptide separation rates, sensitive and ultrafast MS, and sophisticated data analysis strategies are essential to successful LF‐SCP analyses. By utilizing and combining these high‐end methodologies the up to 2000 proteins were identified from HeLa cells, which is expected to improve even further. Current limitations and challenges for LF‐SCP are associated with sample throughput and sensitivity, which are currently being tackled by ultrafast LC gradients and the use of MBR to libraries of hundreds to thousands of cells with great success. Including MBR more than 2500 proteins quantified were reported in a recent technote from Evosep [113]. With several single cell omics technology coming of age, the perspective of realizing multi‐omics analyses from an individual cells becomes extremely attractive and has already been realized for transcriptomics and proteomics using the nanodroplet splitting for linked‐multimodal Investigations of trace samples (nanoSPLITS) technology [114]. In a very innovative work of Mahdessian et al. [115], single cell proteogenomics, although not from the very same cell yet, was successfully applied to generate a spatiotemporal map of human proteomic heterogeneity at subcellular resolution. The data was correlated with single‐cell transcriptomics and cell cycle state information. The authors found that cell cycle progression explains less than half of all cellular heterogeneity and conclude that post translational regulation is predominant over regulation by transcriptomic cycling. Deep visual proteomics, as demonstrated by the Mann and coworkers [43], is another potentially groundbreaking approach to improve our understanding of spatial proteomics in future. AI driven image analysis of different cell phenotypes is combined with single cell or even single nucleus laser microdissection for analysis by MS. This allows to preserve spatial context and map proteome abundances to (sub‐)cellular phenotypes. The authors of that study justifiably claim that this might be a key technology for future research on cancer progression, diagnostics, and drug development. Arguably deep visual proteomics could be extended to any system that can be microscopically imaged. Combining SCPs with other omic‐techniques like metabolomics or lipidomics could be potentially an even more relevant approach to comprehensively map phenotype‐defining molecular signatures. Furthermore, classical MS‐based SCP might be combined with high potential techniques such as mass cytometry and imaging mass cytometry in future as already impressively demonstrated in linking individual breast epithelial cells to age, parity, and BRCA2 status [116] and as summarized in an excellent review elsewhere [117]. We therefore believe that LF SCP represents an indispensable approach in the SCP field to assess cellular heterogeneity to identify rare subpopulations with high relevance in biology, which offers specific benefits over multiplexed SCP or CyTOF with an exciting outlook for future applications.

## AUTHOR CONTRIBUTIONS

Manuel Matzinger and Rupert L. Mayer conceptualized and wrote the manuscript.

## CONFLICT OF INTEREST

The authors declare no conflict of interest.

## Data Availability

Raw data can be accessed free of charge via the ProteomeXchange Consortium in the PRIDE partner repository with the dataset identifier PXD039208.

## References

[1] Vegliante, R. , Pastushenko, I. , & Blanpain, C. (2022). Deciphering functional tumor states at single‐cell resolution. The EMBO Journal, 41, e109221. 34918370 10.15252/embj.2021109221 PMC8762559

[2] Eze, U. C. , Bhaduri, A. , Haeussler, M. , & Nowakowski, T. J. , Kriegstein, A. R. (2021). Single‐cell atlas of early human brain development highlights heterogeneity of human neuroepithelial cells and early radial glia. Nature Neuroscience, 24, 584–594. 33723434 10.1038/s41593-020-00794-1 PMC8012207

[3] Giladi, A. , Paul, F. , Herzog, Y. , Lubling, Y. , Weiner, A. , Yofe, I. , Jaitin, D. , Cabezas‐Wallscheid, N. , Dress, R. , Ginhoux, F. , Trumpp, A. , Tanay, A. , & Amit, I. (2018). Single‐cell characterization of haematopoietic progenitors and their trajectories in homeostasis and perturbed haematopoiesis. Nature Cell Biology, 20, 836–846. 29915358 10.1038/s41556-018-0121-4

[4] Wilson, N. K. , Kent, D. G. , Buettner, F. , Shehata, M. , Macaulay, I. C. , Calero‐Nieto, F. J. , Sánchez Castillo, M. , Oedekoven, C. A. , Diamanti, E. , Schulte, R. , Ponting, C. P. , Voet, T. , Caldas, C. , Stingl, J. , Green, A. R. , Theis, F. J. , & Göttgens, B. (2015). Combined single‐cell functional and gene expression analysis resolves heterogeneity within stem cell populations. Cell Stem Cell, 16, 712–724. 26004780 10.1016/j.stem.2015.04.004 PMC4460190

[5] Moignard, V. , Macaulay, I. C. , Swiers, G. , Buettner, F. , Schütte, J. , Calero‐Nieto, F. J. , Kinston, S. , Joshi, A. , Hannah, R. , Theis, F. J. , Jacobsen, S. E. , De Bruijn, M. F. , & Göttgens, B. (2013). Characterization of transcriptional networks in blood stem and progenitor cells using high‐throughput single‐cell gene expression analysis. Nature Cell Biology, 15, 363–372. 23524953 10.1038/ncb2709 PMC3796878

[6] Lombard‐Banek, C. , Moody, S. A. , & Nemes, P. (2016). Single‐cell mass spectrometry for discovery proteomics: Quantifying translational cell heterogeneity in the 16‐cell frog (Xenopus) embryo. Angewandte Chemie International Edition, 55, 2454–2458. 26756663 10.1002/anie.201510411 PMC4755155

[7] Virant‐Klun, I. , Leicht, S. , Hughes, C. , & Krijgsveld, J. (2016). Identification of maturation‐specific proteins by single‐cell proteomics of human oocytes. Molecular & Cellular Proteomics, 15, 2616–2627. 27215607 10.1074/mcp.M115.056887 PMC4974340

[8] Furtwängler, B. , Üresin, N. , Motamedchaboki, K. , Huguet, R. , Lopez‐Ferrer, D. , Zabrouskov, V. , Porse, B. T. , & Schoof, E. M. (2022). Real‐time search‐assisted acquisition on a tribrid mass spectrometer improves coverage in multiplexed single‐cell proteomics. Molecular & Cellular Proteomics, 21, 100219. 35219906 10.1016/j.mcpro.2022.100219 PMC8961214

[9] Woo, J. , Clair, G. C. , Williams, S. M. , Feng, S. , Tsai, C.‐F. , Moore, R. J. , Chrisler, W. B. , Smith, R. D. , Kelly, R. T. , Pasa‐Tolic, L. , Ansong, C. , & Zhu, Y. (2022). Three‐dimensional feature matching improves coverage for single‐cell proteomics based on ion mobility filtering. Cell Systems, 13, 426–434.e4. 35298923 10.1016/j.cels.2022.02.003 PMC9119937

[10] Végvári, Á. , Rodriguez, J. E. , & Zubarev, R. A. (2022). Single‐cell chemical proteomics (SCCP) interrogates the timing and heterogeneity of cancer cell commitment to death. Analytical Chemistry, 94, 9261–9269. 35731985 10.1021/acs.analchem.2c00413 PMC9260713

[11] Matzinger, M. , Mueller, E. , Duernberger, G. , Pichler, P. , & Mechtler, K. (2023). Robust and easy‐to‐use one pot workflow for label free single cell proteomics. Analytical Chemistry, 10.1021/acs.analchem.2c05022 PMC9996606 36802514

[12] Ctortecka, C. , Hartlmayr, D. , Seth, A. , Mendjan, S. , Tourniaire, G. , & Mechtler, K. (2022). An automated workflow for multiplexed single‐cell proteomics sample preparation at unprecedented sensitivity. 2021.04.14.439828. 10.1101/2021.04.14.439828 PMC10684380 37839701

[13] Derks, J. , Leduc, A. , Wallmann, G. , Huffman, R. G. , Willetts, M. , Khan, S. , Specht, H. , Ralser, M. , Demichev, V. , & Slavov, N. (2022). Increasing the throughput of sensitive proteomics by plexDIA. Nature Biotechnology, 50–59. 10.1038/s41587-022-01389-w PMC9839897 35835881

[14] Ctortecka, C. , Stejskal, K. , Krššáková, G. , Mendjan, S. , & Mechtler, K. (2022). Quantitative accuracy and precision in multiplexed single‐cell proteomics. Analytical Chemistry, 94, 2434–2443. 34967612 10.1021/acs.analchem.1c04174 PMC8829824

[15] Schoof, E. M. , Furtwängler, B. , Üresin, N. , Rapin, N. , Savickas, S. , Gentil, C. , Lechman, E. , Keller, U. A. D. , Dick, J. E. , & Porse, Bo T. (2021). Quantitative single‐cell proteomics as a tool to characterize cellular hierarchies. Nature Communication, 12, 3341. 10.1038/s41467-021-23667-y PMC8185083 34099695

[16] Budnik, B. , Levy, E. , Harmange, G. , & Slavov, N. (2018). SCoPE‐MS: Mass spectrometry of single mammalian cells quantifies proteome heterogeneity during cell differentiation. Genome Biology, 19, 161. 30343672 10.1186/s13059-018-1547-5 PMC6196420

[17] Yi, L. , Tsai, C.‐F. , Dirice, E. , Swensen, A. C. , Chen, J. , Shi, T. , Gritsenko, M. A. , Chu, R. K. , Piehowski, P. D. , Smith, R. D. , Rodland, K. D. , Atkinson, M. A. , Mathews, C. E. , Kulkarni, R. N. , Liu, T. , & Qian, W.‐J. (2019). Boosting to amplify signal with isobaric labeling (BASIL) strategy for comprehensive quantitative phosphoproteomic characterization of small populations of cells. Analytical Chemistry, 91, 5794–5801. 30843680 10.1021/acs.analchem.9b00024 PMC6596310

[18] Tan, Z. , Yi, X. , Carruthers, N. J. , Stemmer, P. M. , & Lubman, D. M. (2019). Single amino acid variant discovery in small numbers of cells. Journal of Proteome Research, 18, 417–425. 30404448 10.1021/acs.jproteome.8b00694 PMC6465122

[19] Petelski, A. A. , Emmott, E. , Leduc, A. , Huffman, R. G. , Specht, H. , Perlman, D. H. , & Slavov, N. (2021). Multiplexed single‐cell proteomics using SCoPE2. Nature Protocols, 16, 5398–5425. 34716448 10.1038/s41596-021-00616-z PMC8643348

[20] Li, J. , Cai, Z. , Bomgarden, R. D. , Pike, I. , Kuhn, K. , Rogers, J. C. , Roberts, T. M. , Gygi, S. P. , & Paulo, J. A. (2021). TMTpro‐18plex: The expanded and complete set of TMTpro reagents for sample multiplexing. Journal of Proteome Research, 20, 2964–2972. 33900084 10.1021/acs.jproteome.1c00168 PMC8210943

[21] Werner, T. , Becher, I. , Sweetman, G. , Doce, C. , Savitski, M. M. , & Bantscheff, M. (2012). High‐resolution enabled TMT 8‐plexing. Analytical Chemistry, 84, 7188–7194. 22881393 10.1021/ac301553x

[22] Shirran, S. L. , & Botting, C. H. (2010). A comparison of the accuracy of iTRAQ quantification by nLC‐ESI MSMS and nLC‐MALDI MSMS methods. Journal of Proteomics, 73, 1391–1403. 20230925 10.1016/j.jprot.2010.03.003 PMC2880794

[23] Karp, N. A. , Huber, W. , Sadowski, P. G. , Charles, P. D. , Hester, S. V. , & Lilley, K. S. (2010). Addressing accuracy and precision issues in iTRAQ quantitation. Molecular & Cellular Proteomics, 9, 1885–1897. 20382981 10.1074/mcp.M900628-MCP200 PMC2938101

[24] Ow, S. Y. , Salim, M. , Noirel, J. , Evans, C. , Rehman, I. , & Wright, P. C. (2009). iTRAQ underestimation in simple and complex mixtures: “The Good, the Bad and the Ugly”. Journal of Proteome Research, 8, 5347–5355. 19754192 10.1021/pr900634c

[25] Savitski, M. M. , Mathieson, T. , Zinn, N. , Sweetman, G. , Doce, C. , Becher, I. , Pachl, F. , Kuster, B. , & Bantscheff, M. (2013). Measuring and managing ratio compression for accurate iTRAQ/TMT quantification. Journal of Proteome Research, 12, 3586–3598. 23768245 10.1021/pr400098r

[26] Kelstrup, C. D. , Aizikov, K. , Batth, T. S. , Kreutzman, A. , Grinfeld, D. , Lange, O. , Mourad, D. , Makarov, A. A. , & Olsen, J. V. (2018). Limits for resolving isobaric tandem mass tag reporter ions using phase‐constrained spectrum deconvolution. Journal of Proteome Research, 17, 4008–4016. 30220210 10.1021/acs.jproteome.8b00381

[27] Ctortecka, C. , Krššáková, G. , Stejskal, K. , Penninger, J. M. , Mendjan, S. , Mechtler, K. , & Stadlmann, J. (2021). Comparative proteome signatures of trace samples by multiplexed data‐independent acquisition. 10.1101/2021.02.11.430601 PMC8717550 34793982

[28] Li, F. , Schmerberg, C. M. , & Ji, Q. C. (2009). Accelerated tryptic digestion of proteins in plasma for absolute quantitation using a protein internal standard by liquid chromatography/tandem mass spectrometry. Rapid Communications in Mass Spectrometry, 23, 729–732. 19191257 10.1002/rcm.3926 PMC3016225

[29] Liang, Y. , Acor, H. , Mccown, M. A. , Nwosu, A. J. , Boekweg, H. , Axtell, N. B. , Truong, T. , Cong, Y. , Payne, S. H. , & Kelly, R. T. (2021). Fully automated sample processing and analysis workflow for low‐input proteome profiling. Analytical Chemistry, 93, 1658–1666. 33352054 10.1021/acs.analchem.0c04240 PMC8140400

[30] Li, Z.‐Y. , Huang, M. , Wang, X.‐K. , Zhu, Y. , Li, J.‐S. , Wong, C. C. L. , & Fang, Q. (2018). Nanoliter‐scale oil‐air‐droplet chip‐based single cell proteomic analysis. Analytical Chemistry, 90, 5430–5438. 29551058 10.1021/acs.analchem.8b00661

[31] Alexovic, M. , Sabo, J. , & Longuespée, R. (2021). Automation of single‐cell proteomic sample preparation. Proteomics, 21, 2100198. 10.1002/pmic.202100198 34570421

[32] Wu, R. , Xing, S. , Badv, M. , Didar, T. F. , & Lu, Y. (2019). Step‐wise assessment and optimization of sample handling recovery yield for nanoproteomic analysis of 1000 mammalian cells. Analytical Chemistry, 91, 10395–10400. 31318197 10.1021/acs.analchem.9b02092

[33] Volpe, P. , & Eremenko‐Volpe, T. (1970). Quantitative studies on cell proteins in suspension cultures. European Journal of Biochemistry, 12, 195–200. 5434280 10.1111/j.1432-1033.1970.tb00837.x

[34] Matsumoto, C. , Shao, X. , Bogosavljevic, M. , Chen, L. , & Gao, Y. (2022). Automated container‐less cell processing method for single‐cell proteomics. 2022.07.26.501646. 10.1101/2022.07.26.501646

[35] Chang, Y.‐H. , Gregorich, Z. R. , Chen, A. J. , Hwang, L. , Guner, H. , Yu, D. , Zhang, J. , & Ge, Y. (2015). New mass‐spectrometry‐compatible degradable surfactant for tissue proteomics. Journal of Proteome Research, 14, 1587–1599. 25589168 10.1021/pr5012679 PMC4384424

[36] Liu, J. , Wang, F. , Mao, J. , Zhang, Z. , Liu, Z. , Huang, G. , Cheng, K. , & Zou, H. (2015). High‐sensitivity N‐glycoproteomic analysis of mouse brain tissue by protein extraction with a mild detergent of N‐dodecyl β‐D‐maltoside. Analytical Chemistry, 87, 2054–2057. 25646822 10.1021/ac504700t

[37] Zhu, Y. , Clair, G. , Chrisler, W. B. , Shen, Y. , Zhao, R. , Shukla, A. K. , Moore, R. J. , Misra, R. S. , Pryhuber, G. S. , Smith, R. D. , Ansong, C. , & Kelly, R. T. (2018). Proteomic analysis of single mammalian cells enabled by microfluidic nanodroplet sample preparation and ultrasensitive NanoLC‐MS. Angewandte Chemie International Edition, 57, 12370–12374. 29797682 10.1002/anie.201802843 PMC6261339

[38] Cong, Y. , Motamedchaboki, K. , Misal, A. , Liang, Y. , Guise, A. J. , Truong, T. , Huguet, R. , Plowey, E. D. , Zhu, Y. , Lopez‐Ferrer, D. , & Kelly, R. T. (2021). Ultrasensitive single‐cell proteomics workflow identifies >1000 protein groups per mammalian cell. Chemical Science, 12, 1001–1006. 10.1039/d0sc03636f PMC8178986 34163866

[39] Brunner, A.‐D. , Thielert, M. , Vasilopoulou, C. , Ammar, C. , Coscia, F. , Mund, A. , Hoerning, O. B. , Bache, N. , Apalategui, A. , Lubeck, M. , Richter, S. , Fischer, D. S. , Raether, O. , Park, M. A. , Meier, F. , Theis, F. J. , & Mann, M. (2022). Ultra‐high sensitivity mass spectrometry quantifies single‐cell proteome changes upon perturbation. Molecular Systems Biology, 18, e10798. 35226415 10.15252/msb.202110798 PMC8884154

[40] Specht, H. , Harmange, G. , Perlman, D. H. , Emmott, E. , Niziolek, Z. , Budnik, B. , & Slavov, N. (2018). Automated sample preparation for high‐throughput single‐cell proteomics. 399774. 10.1101/399774

[41] Leduc, A. , Huffman, R. G. , Cantlon, J. , Khan, S. , & Slavov, N. (2022). Exploring functional protein covariation across single cells using nPOP. Genoome Biology, 23, 261. 10.1186/s13059-022-02817-5 PMC9756690 36527135

[42] Shao, X. , Wang, X. , Guan, S. , Lin, H. , Yan, G. , Gao, M. , Deng, C. , & Zhang, X. (2018). Integrated proteome analysis device for fast single‐cell protein profiling. Analytical Chemistry, 90, 14003–14010. 30375851 10.1021/acs.analchem.8b03692

[43] Johnson, K. R. , Gao, Y. , Gregus, M. , & Ivanov, A. R. (2022). On‐capillary cell lysis enables top‐down proteomic analysis of single mammalian cells by CE‐MS/MS. Analytical Chemistry, 94, 14358–14367. 36194750 10.1021/acs.analchem.2c03045 PMC10118848

[44] Mund, A. , Coscia, F. , Kriston, A. , Hollandi, R. , Kovács, F. , Brunner, A.‐D. , Migh, E. , Schweizer, L. , Santos, A. , Bzorek, M. , Naimy, S. , Rahbek‐Gjerdrum, L. M. , Dyring‐Andersen, B. , Bulkescher, J. , Lukas, C. , Eckert, M. A. , Lengyel, E. , Gnann, C. , Lundberg, E. , … Mann, M. (2022). Deep visual proteomics defines single‐cell identity and heterogeneity. Nature Biotechnology, 40, 1231–1240. 10.1038/s41587-022-01302-5 PMC9371970 35590073

[45] Petrosius, V. , Aragon‐Fernandez, P. , Uresin, N. , Phlairaharn, T. , Furtwangler, B. , de Beeck, J. , Thomsen, S. F. , auf dem Keller, U. , Porse, B. T. , & Schoof, E. M. (2022). Enhancing single‐cell proteomics through tailored Data‐Independent Acquisition and micropillar array‐based chromatography. 2022.11.29.518366. 10.1101/2022.11.29.518366

[46] Shishkova, E. , Hebert, A. S. , & Coon, J. J. (2016). Now, more than ever, proteomics needs better chromatography. Cell Systems, 3, 321–324. 27788355 10.1016/j.cels.2016.10.007 PMC5448283

[47] Gatlin, C. L. , Kleemann, G. R. , Hays, L. G. , Link, A. J. , & Yates, J. R. (1998). Protein identification at the low femtomole level from silver‐stained gels using a new fritless electrospray interface for liquid chromatography–microspray and nanospray mass spectrometry. Analytical Biochemistry, 263, 93–101. 9750149 10.1006/abio.1998.2809

[48] Wilm, M. , & Mann, M. (1996). Analytical properties of the nanoelectrospray ion source. Analytical Chemistry, 68, 1–8. 8779426 10.1021/ac9509519

[49] Zheng, R. , Pynn, C. , Matzinger, M. , Mechtler, K. , Makarov, A. , Decrop, W. , Valenta, A. , & Samonig, M. (2022). A high‐sensitivity high‐throughput LCMS platform for single‐cell proteomics and low sample amount analysis. https://www.analyteguru.com/t5/Scientific‐Library/High‐sensitivity‐and‐throughput‐LCMS‐for‐single‐cell‐proteomics/ta‐p/19598

[50] Lenco, J. , Jadeja, S. , Naplekov, D. K. , Krokhin, O. V. , Khalikova, M. A. , Chocholous, P. , Urban, J. , Broeckhoven, K. , Nováková, L. , & Svec, F. (2022). Reversed‐phase liquid chromatography of peptides for bottom‐up proteomics: A tutorial. Journal of Proteome Research, 2846–2892. 10.1021/acs.jproteome.2c00407 36355445

[51] Villalobos Solis, M. I. , Giannone, R. J. , Hettich, R. L. , & Abraham, P. E. (2019). Exploiting the dynamic relationship between peptide separation quality and peptide coisolation in a multiple‐peptide matches‐per‐spectrum approach offers a strategy to optimize bottom‐up proteomics throughput and depth. Analytical Chemistry, 91, 7273–7279. 31075198 10.1021/acs.analchem.9b00819

[52] Xiang, P. , Zhu, Y. , Yang, Y. , Zhao, Z. , Williams, S. M. , Moore, R. J. , Kelly, R. T. , Smith, R. D. , & Liu, S. (2020). Picoflow liquid chromatography–mass spectrometry for ultrasensitive bottom‐up proteomics using 2‐µm‐i.d. open tubular columns. Analytical Chemistry, 92, 4711–4715. 32208662 10.1021/acs.analchem.9b05639 PMC7279521

[53] Geyer, P. E. , Kulak, N. A. , Pichler, G. , Holdt, L. M. , Teupser, D. , & Mann, M. (2016). Plasma proteome profiling to assess human health and disease. Cell Systems, 2, 185–195. 27135364 10.1016/j.cels.2016.02.015

[54] Geyer, P. E. , Wewer Albrechtsen, N. J. , Tyanova, S. , Grassl, N. , Iepsen, E. W. , Lundgren, J. , Madsbad, S. , Holst, J. J. , Torekov, S. S. , & Mann, M. (2016). Proteomics reveals the effects of sustained weight loss on the human plasma proteome. Molecular Systems Biology, 12, 901. 28007936 10.15252/msb.20167357 PMC5199119

[55] Ivanov, M. V. , Bubis, J. A. , Gorshkov, V. , Tarasova, I. A. , Levitsky, L. I. , Lobas, A. A. , Solovyeva, E. M. , Pridatchenko, M. L. , Kjeldsen, F. , & Gorshkov, M. V. (2020). DirectMS1: MS/MS‐free identification of 1000 proteins of cellular proteomes in 5 minutes. Analytical Chemistry, 92, 4326–4333. 32077687 10.1021/acs.analchem.9b05095

[56] Ivanov, M. V. , Bubis, J. A. , Gorshkov, V. , Tarasova, I. A. , Levitsky, L. I. , Solovyeva, E. M. , Lipatova, A. V. , Kjeldsen, F. , & Gorshkov, M. V. (2022). DirectMS1Quant: Ultrafast quantitative proteomics with MS/MS‐free mass spectrometry. Analytical Chemistry, 94, 13068–13075. 36094425 10.1021/acs.analchem.2c02255

[57] Bache, N. , Geyer, P. E. , Bekker‐Jensen, D. B. , Hoerning, O. , Falkenby, L. , Treit, P. V. , Doll, S. , Paron, I. , Müller, J. B. , Meier, F. , Olsen, J. V. , Vorm, O. , & Mann, M. (2018). A novel LC system embeds analytes in pre‐formed gradients for rapid, ultra‐robust proteomics. Molecular & Cellular Proteomics, 17, 2284–2296. 30104208 10.1074/mcp.TIR118.000853 PMC6210218

[58] Webber, K. G. I. , Truong, T. , Johnston, S. M , Zapata, S. E. , Liang, Y. , Davis, J. M. , Buttars, A. D. , Smith, F. B. , Jones, H. E. , Mahoney, A. C. , Carson, R. H. , Nwosu, A. J. , Heninger, J. L. , Liyu, A. V. , Nordin, G. P. , Zhu, Y. , & Kelly, R. T. (2022). Label‐free profiling of up to 200 single‐cell proteomes per day using a dual‐column nanoflow liquid chromatography platform. Analytical Chemistry, 94, 6017–6025. 35385261 10.1021/acs.analchem.2c00646 PMC9356711

[59] Orton, D. J. , Wall, M. J. , & Doucette, A. A. (2013). Dual LC–MS platform for high‐throughput proteome analysis. Journal of Proteome Research, 12, 5963–5970. 24090060 10.1021/pr400738a

[60] Vissers, J. P. C. , Blackburn, R. K. , & Moseley, M. A. (2002). A novel interface for variable flow nanoscale LC/MS/MS for improved proteome coverage. Journal of the American Society for Mass Spectrometry, 13, 760–771. 12148801 10.1016/S1044-0305(02)00418-X

[61] Phlairaharn, T. , Grégoire, S. , Woltereck, L. R. , Petrosius, V. , Furtwängler, B. , Searle, B. C. , & Schoof, E. M. (2022). High sensitivity limited material proteomics empowered by data‐independent acquisition on linear ion traps. Journal of Proteome Research, 21, 2815–2826. 36287219 10.1021/acs.jproteome.2c00376

[62] Sorensen, M. J. , Anderson, B. G. , & Kennedy, R. T. (2020). Liquid chromatography above 20,000 PSI. TrAC Trends in Analytical Chemistry, 124, 115810. 32382203 10.1016/j.trac.2020.115810 PMC7204529

[63] Hsieh, E. J. , Bereman, M. S. , Durand, S. , Valaskovic, G. A. , & Maccoss, M. J. (2013). Effects of column and gradient lengths on peak capacity and peptide identification in nanoflow LC‐MS/MS of complex proteomic samples. Journal of the American Society for Mass Spectrometry, 24, 148–153. 23197307 10.1007/s13361-012-0508-6 PMC3554873

[64] Giesche, H. , Unger, K. K. , Esser, U. , Eray, B. , Trüdinger, U. , & Kinkel, J. N. (1989). Packing technology, column bed structure and chromatographic performance of 1‐2‐µm non‐porous silicas in high‐performance liquid chromatography. Journal of Chromatography A, 465, 39–57.

[65] Gzil, P. , Vervoort, N. , Baron, G. V. , & Desmet, G. (2003). Advantages of perfectly ordered 2‐d porous pillar arrays over packed bed columns for LC separations: A theoretical analysis. Analytical Chemistry, 75, 6244–6250. 14616008 10.1021/ac034345m

[66] De Malsche, W. , Op De Beeck, J. , De Bruyne, S. , Gardeniers, H. , & Desmet, G. (2012). Realization of 1 × 106 theoretical plates in liquid chromatography using very long pillar array columns. Analytical Chemistry, 84, 1214–1219. 22208432 10.1021/ac203048n

[67] Gritti, F. , & Guiochon, G. (2013). Perspectives on the evolution of the column efficiency in liquid chromatography. Analytical Chemistry, 85, 3017–3035. 23414563 10.1021/ac3033307

[68] Horvath, C. G. , & Lipsky, S. R. (1969). Rapid analysis of ribonucleosides and bases at the picomole level using pellicular cation exchange resin in narrow bore columns. Analytical Chemistry, 41, 1227–1234. 5797315 10.1021/ac60279a024

[69] Mayer, R. L. , Matzinger, M. , Schmücker, A. , Stejskal, K. , Krššáková, G. , Berger, F. , & Mechtler, K. (2022). Wide window acquisition and AI‐based data analysis to reach deep proteome coverage for a wide sample range, including single cell proteomic inputs. 2022.09.01.506203. 10.1101/2022.09.01.506203

[70] Stejskal, K. , Op De Beeck, J. , Dürnberger, G. , Jacobs, P. , & Mechtler, K. (2021). Ultrasensitive NanoLC‐MS of subnanogram protein samples using second generation micropillar array LC technology with Orbitrap Exploris 480 and FAIMS PRO. Analytical Chemistry, 93, 8704–8710. 34137250 10.1021/acs.analchem.1c00990 PMC8253486

[71] Thompson, A. , Wölmer, N. , Koncarevic, S. , Selzer, S. , Böhm, G. , Legner, H. , Schmid, P. , Kienle, S. , Penning, P. , Höhle, C. , Berfelde, A. , Martinez‐Pinna, R. , Farztdinov, V. , Jung, S. , Kuhn, K. , & Pike, I. (2019). TMTpro: Design, synthesis, and initial evaluation of a proline‐based isobaric 16‐Plex tandem mass tag reagent set. Analytical Chemistry, 91, 15941–15950. 31738517 10.1021/acs.analchem.9b04474

[72] Demichev, V. , Messner, C. B. , Vernardis, S. I. , Lilley, K. S. , & Ralser, M. (2020). DIA‐NN: Neural networks and interference correction enable deep proteome coverage in high throughput. Nature Methods, 17, 41–44. 31768060 10.1038/s41592-019-0638-x PMC6949130

[73] Huang, T. , Bruderer, R. , Muntel, J. , Xuan, Y. , Vitek, O. , & Reiter, L. (2020). Combining precursor and fragment information for improved detection of differential abundance in data independent acquisition. Molecular & Cellular Proteomics, 19, 421–430. 31888964 10.1074/mcp.RA119.001705 PMC7000113

[74] Xuan, Y. , Bateman, N. W. , Gallien, S. , Goetze, S. , Zhou, Y. , Navarro, P. , Hu, M. , Parikh, N. , Hood, B. L. , Conrads, K. A. , Loosse, C. , Kitata, R. B. , Piersma, S. R. , Chiasserini, D. , Zhu, H. , Hou, G. , Tahir, M. , Macklin, A. , Khoo, A. , … Conrads, T. P. (2020). Standardization and harmonization of distributed multi‐center proteotype analysis supporting precision medicine studies. Nature Communication, 11, 5248. 10.1038/s41467-020-18904-9 PMC7568553 33067419

[75] Borràs, E. , Pastor, O. , & Sabidó, E. (2021). Use of linear ion traps in data‐independent acquisition methods benefits low‐input proteomics. Analytical Chemistry, 93, 11649–11653. 34404205 10.1021/acs.analchem.1c01885

[76] Hebert, A. S. , Prasad, S. , Belford, M. W. , Bailey, D. J. , McAlister, G. C. , Abbatiello, S. E. , Huguet, R. , Wouters, E. R. , Dunyach, J.‐J. , Brademan, D. R. , Westphall, M. S. , & Coon, J. J. (2018). Comprehensive single‐shot proteomics with FAIMS on a hybrid orbitrap mass spectrometer. Analytical Chemistry, 90, 9529–9537. 29969236 10.1021/acs.analchem.8b02233 PMC6145172

[77] Barnett, D. A. , Ells, B. , Guevremont, R. , & Purves, R. W. (2002). Application of ESI‐FAIMS‐MS to the analysis of tryptic peptides. Journal of the American Society for Mass Spectrometry, 13, 1282–1291. 12443018 10.1016/S1044-0305(02)00527-5

[78] Bekker‐Jensen, D. B. , Martínez‐Val, A. , Steigerwald, S. , Rüther, P. , Fort, K. L. , Arrey, T. N. , Harder, A. , Makarov, A. , & Olsen, J. V. (2020). A compact quadrupole‐orbitrap mass spectrometer with FAIMS interface improves proteome coverage in short LC gradients. Molecular & Cellular Proteomics, 19, 716–729. 32051234 10.1074/mcp.TIR119.001906 PMC7124470

[79] Gregus, M. , Kostas, J. C. , Ray, S. , Abbatiello, S. E. , & Ivanov, A. R. (2020). Improved sensitivity of ultralow flow LC–MS‐based proteomic profiling of limited samples using monolithic capillary columns and FAIMS technology. Analytical Chemistry, 92, 14702–14712. 33054160 10.1021/acs.analchem.0c03262 PMC7934643

[80] Ridgeway, M. E. , Lubeck, M. , Jordens, J. , Mann, M. , & Park, M. A. (2018). Trapped ion mobility spectrometry: A short review. International Journal of Mass Spectrometry, 425, 22–35.

[81] Brzhozovskiy, A. , Kononikhin, A. , Bugrova, A. E. , Kovalev, G. I. , Schmit, P.‐O. , Kruppa, G. , Nikolaev, E. N. , & Borchers, C. H. (2022). The parallel reaction monitoring‐parallel accumulation–serial fragmentation (prm‐PASEF) approach for multiplexed absolute quantitation of proteins in human plasma. Analytical Chemistry, 94, 2016–2022. 35040635 10.1021/acs.analchem.1c03782

[82] Krisp, C. , Seth, A. , Schmit, P.‐O. , Hartlmayr, D. , Srikumar, T. , Krieger, J. , Lubeck, M. , Tourniare, G. , & Kruppa, G. (2022). Label‐free single cell proteomics workflow with the cellenONE platform and the timsTOF SCP. *TechNote‐Bruker Cellenion*. https://www.cellenion.com/wp‐content/uploads/2022/03/25‐App‐Note‐Label‐Free‐single‐cell‐proteomics‐with‐Brukers‐timsTOF‐SCP.pdf retrieved 12/13/2022)

[83] Kelstrup, C. D. , Bekker‐Jensen, D. B. , Arrey, T. N. , Hogrebe, A. , Harder, A. , & Olsen, J. V. (2018). Performance Evaluation of the Q Exactive HF‐X for shotgun proteomics. Journal of Proteome Research, 17, 727–738. 29183128 10.1021/acs.jproteome.7b00602

[84] Plank, M. J. (2022). Modern data acquisition approaches in proteomics based on dynamic instrument control. Journal of Proteome Research, 21, 1209–1217. 35362319 10.1021/acs.jproteome.2c00096

[85] Wichmann, C. , Meier, F. , Virreira Winter, S. , Brunner, A.‐D. , Cox, J. , & Mann, M. (2019). MaxQuant.Live enables global targeting of more than 25,000 peptides. Molecular & Cellular Proteomics, 18, 982–994. 10.1074/mcp.TIR118.001131 PMC6495250 30755466

[86] Huffman, R. G. , Leduc, A. , Wichmann, C. , Gioia, M. , Borriello, F. , Specht, H. , Derks, J. , Khan, s. , Emmot, E. , Petelski, A. A. , Perlmann, D. H. , Cox, J. , Zanoni, I. , & Slavov, N. (2022). Prioritized single‐cell proteomics reveals molecular and functional polarization across primary macrophages. 10.1101/2022.03.16.484655

[87] Ivanov, M. V. , Bubis, J. A. , Gorshkov, V. , Abdrakhimov, D. A. , Kjeldsen, F. , & Gorshkov, M. V. (2021). Boosting MS1‐only proteomics with machine learning allows 2000 protein identifications in single‐shot human proteome analysis using 5 min HPLC gradient. Journal of Proteome Research, 20, 1864–1873. 33720732 10.1021/acs.jproteome.0c00863

[88] Solovyeva, E. M. , Bubis, J. A. , Tarasova, I. A. , Lobas, A. A. , Ivanov, M. V. , Nazarov, A. A. , Shutkov, I. A. , & Gorshkov, M. V. (2022). On the feasibility of using an ultra‐fast DirectMS1 method of proteome‐wide analysis for searching drug targets in chemical proteomics. Biochemistry (Moscow), 87, 1342–1353. 36509723 10.1134/S000629792211013X

[89] Kong, W. , Hui, H. W. H. , Peng, H. , & Goh, W. W. B. Dealing with missing values in proteomics data. Proteomics, n/a, 2200092. 10.1002/pmic.202200092 36349819

[90] Perez‐Riverol, Y. , Csordas, A. , Bai, J. , Bernal‐Llinares, M. , Hewapathirana, S. , Kundu, D. J. , Inuganti, A. , Griss, J. , Mayer, G. , Eisenacher, M. , Pérez, E. , Uszkoreit, J. , Pfeuffer, J. , Sachsenberg, T. , Yilmaz, S. , Tiwary, S. , Cox, J. , Audain, E. , Walzer, M. , … Vizcaíno, J. A. (2019). The PRIDE database and related tools and resources in 2019: Improving support for quantification data. Nucleic Acids Research, 47, D442–D450. 30395289 10.1093/nar/gky1106 PMC6323896

[91] Doblmann, J. , Dusberger, F. , Imre, R. , Hudecz, O. , Stanek, F. , Mechtler, K. , & Dürnberger, G. (2019). apQuant: Accurate label‐free quantification by quality filtering. Journal of Proteome Research, 18, 535–541. 30351950 10.1021/acs.jproteome.8b00113

[92] Eng, J. K. , Mccormack, A. L. , & Yates, J. R. (1994). An approach to correlate tandem mass spectral data of peptides with amino acid sequences in a protein database. Journal of the American Society for Mass Spectrometry, 5, 976–989. 24226387 10.1016/1044-0305(94)80016-2

[93] Reiter, L. , Rinner, O. , Picotti, P. , Hüttenhain, R. , Beck, M. , Brusniak, M.‐Y. , Hengartner, M. O. , & Aebersold, R. (2011). mProphet: Automated data processing and statistical validation for large‐scale SRM experiments. Nature Methods, 8, 430–435. 21423193 10.1038/nmeth.1584

[94] Cox, J. , & Mann, M. (2008). MaxQuant enables high peptide identification rates, individualized p.p.b.‐range mass accuracies and proteome‐wide protein quantification. Nature Biotechnology, 26, 1367–1372. 10.1038/nbt.1511 19029910

[95] Cox, J. , Neuhauser, N. , Michalski, A. , Scheltema, R. A. , Olsen, J. V. , & Mann, M. (2011). Andromeda: A peptide search engine integrated into the MaxQuant environment. Journal of Proteome Research, 10, 1794–1805. 21254760 10.1021/pr101065j

[96] Tran, N. H. , Rahman, M. Z , He, L. , Xin, L. , Shan, B. , & Li, M. (2016). Complete de novo assembly of monoclonal antibody sequences. Science Reports, 6, 31730. 10.1038/srep31730 PMC4999880 27562653

[97] Kong, A. T. , Leprevost, F. V. , Avtonomov, D. M. , Mellacheruvu, D. , & Nesvizhskii, A. I. (2017). MSFragger: Ultrafast and comprehensive peptide identification in mass spectrometry–based proteomics. Nature Methods, 14, 513–520. 28394336 10.1038/nmeth.4256 PMC5409104

[98] Boekweg, H. , Van Der Watt, D. , Truong, T. , Johnston, S. M , Guise, A. J. , Plowey, E. D. , Kelly, R. T. , & Payne, S. H. (2022). Features of peptide fragmentation spectra in single‐cell proteomics. Journal of Proteome Research, 21, 182–188. 34920664 10.1021/acs.jproteome.1c00670

[99] Zolg, D. P. , Gessulat, S. , Paschke, C. , Graber, M. , Rathke‐Kuhnert, M. , Seefried, F. , Fitzemeier, K. , Berg, F. , Lopez‐Ferrer, D. , Horn, D. , Henrich, C. , Huhmer, A. , Delanghe, B. , & Frejno, M. (2021). INFERYS rescoring: Boosting peptide identifications and scoring confidence of database search results. Rapid Communications in Mass Spectrometry, n/a, e9128. 10.1002/rcm.9128 34015160

[100] Kalxdorf, M. , Müller, T. , Stegle, O. , & Krijgsveld, J. (2021). IceR improves proteome coverage and data completeness in global and single‐cell proteomics. Nature Communication, 12, 4787. 10.1038/s41467-021-25077-6 PMC8352929 34373457

[101] Zhu, Y. , Scheibinger, M. , Ellwanger, D. C. , Krey, J. F. , Choi, D. , Kelly, R. T. , Heller, S. , & Barr‐Gillespie, P. G. (2019). Single‐cell proteomics reveals changes in expression during hair‐cell development. eLife, 8, e50777. 31682227 10.7554/eLife.50777 PMC6855842

[102] Cox, J. , Hein, M. Y. , Luber, C. A. , Paron, I. , Nagaraj, N. , & Mann, M. (2014). Accurate proteome‐wide label‐free quantification by delayed normalization and maximal peptide ratio extraction, termed MaxLFQ*. Molecular & Cellular Proteomics, 13, 2513–2526. 24942700 10.1074/mcp.M113.031591 PMC4159666

[103] Shen, X. , Shen, S. , Li, J. , Hu, Q. , Nie, L. , Tu, C. , Wang, X. , Poulsen, D. J. , Orsburn, B. C. , Wang, J. , & Qu, J. (2018). IonStar enables high‐precision, low‐missing‐data proteomics quantification in large biological cohorts. Proceedings of the National Academy of Sciences of the United States of America, 115, E4767–E4776. 29743190 10.1073/pnas.1800541115 PMC6003523

[104] Wang, Y. , Lih, T.‐S. M. , Chen, L. , Xu, Y. , Kuczler, M. D. , Cao, L. , Pienta, K. J. , Amend, S. R. , & Zhang, H. (2022). Optimized data‐independent acquisition approach for proteomic analysis at single‐cell level. Clinical Proteomics, 19, 24. 35810282 10.1186/s12014-022-09359-9 PMC9270744

[105] Gebreyesus, S. T. , Siyal, A. A. , Kitata, R. B. , Chen, E. S. , Enkhbayar, B. , Angata, T. , Lin, K.‐I. , Chen, Y.‐J. , & Tu, H.‐L. (2022). Streamlined single‐cell proteomics by an integrated microfluidic chip and data‐independent acquisition mass spectrometry. Nature Communication, 13, 37. 10.1038/s41467-021-27778-4 PMC8748772 35013269

[106] Demichev, V. , Szyrwiel, L. , Yu, F. , Teo, G. C. , Rosenberger, G. , Niewienda, A. , Ludwig, D. , Decker, J. , Kaspar‐Schoenefeld, S. , Lilley, K. S. , Mülleder, M. , Nesvizhskii, A. I. , & Ralser, M. (2022). dia‐PASEF data analysis using FragPipe and DIA‐NN for deep proteomics of low sample amounts. Nature Communication, 13, 3944. 10.1038/s41467-022-31492-0 PMC9270362 35803928

[107] Meier, F. , Brunner, A.‐D. , Frank, M. , Ha, A. , Bludau, I. , Voytik, E. , Kaspar‐Schoenefeld, S. , Lubeck, M. , Raether, O. , Bache, N. , Aebersold, R. , Collins, B. C. , Röst, H. L. , & Mann, M. (2020). diaPASEF: Parallel accumulation–serial fragmentation combined with data‐independent acquisition. Nature Methods, 17, 1229–1236. 33257825 10.1038/s41592-020-00998-0

[108] Szyrwiel, L. , Sinn, L. , Ralser, M. , & Demichev, V. (2022). Slice‐PASEF: Fragmenting all ions for maximum sensitivity in proteomics. 2022.10.31.514544. 10.1101/2022.10.31.514544

[109] Guo, Y. , Cai, L. , Liu, X. , Ma, L. , Zhang, H. , Wang, B. , Qi, Y. , Liu, J. , Diao, F. , Sha, J. , & Guo, X. (2022). Single‐cell quantitative proteomic analysis of human oocyte maturation revealed high heterogeneity in in vitro–matured oocytes. Molecular & Cellular Proteomics, 21, 100267. 35809850 10.1016/j.mcpro.2022.100267 PMC9396076

[110] Walls, M. L. , Hunter, T. , Ryan, J. P. , Keelan, J. A. , Nathan, E. , & Hart, R. J. (2015). In vitro maturation as an alternative to standard in vitro fertilization for patients diagnosed with polycystic ovaries: A comparative analysis of fresh, frozen and cumulative cycle outcomes. Human Reproduction, 30, 88–96. 25355587 10.1093/humrep/deu248

[111] Hu, D. , Dong, X. , Xiong, M. , Xiong, T. , Huang, B. , Zeng, D. , Ai, J. , & Zhang, H. (2015). Can the peak E2/follicle ratio be a quantitative indicator of pregnancy outcomes following assisted reproductive cycles? A retrospective study. International Journal of Clinical and Experimental Medicine, 8, 10964–10970. 26379891 PMC4565274

[112] Li, S. , Su, K. , Zhuang, Z. , Qin, Q. , Gao, L. , Deng, Y. , Liu, X. , Hou, G. , Wang, L. , Hao, P. , Yang, H. , Liu, S. , Zhu, H. , & Ren, Y. (2022). A simple, rapid, and practical method for single‐cell proteomics based on mass‐adaptive coating of synthetic peptides. Science Bulletin, 67, 581–584. 36546118 10.1016/j.scib.2021.12.022

[113] Muggeridge, D. , Dodd, J. , & Ross, M. D. (2021). CD34+ progenitors are predictive of mortality and are associated with physical activity in cardiovascular disease patients. Atherosclerosis, 333, 108–115. 34340831 10.1016/j.atherosclerosis.2021.07.004

[114] Krisp, C. , Lubeck, M. , Kruppa, G. , Almeida, A. , Sandow, J. , Hartlmayr, D. , & Seth, A. (2022). Pushing the boundaries for robust and high‐throughput single cell analysis. *Technote‐Evosep*. https://www.evosep.com/wp‐content/uploads/2022/12/AN‐021A‐Pushing‐the‐boundaries‐for‐robust‐and‐high‐throughput‐single‐cell‐proteomics.pdf (retrieved 12/29/2022)

[115] Fulcher, J. M. , Markillie, L. M. , Mitchell, H. D. , Williams, S. M. , Engbrecht, K. M. , Moore, R. J. , Cantlon‐Bruce, J. , Bagnoli, J. W. , Seth, A. , Paša‐Tolić, L. , & Zhu, Y. (2022). Parallel measurement of transcriptomes and proteomes from same single cells using nanodroplet splitting. 2022.05.17.492137. 10.1101/2022.05.17.492137 PMC11621338 39638780

[116] Mahdessian, D. , Cesnik, A. J. , Gnann, C. , Danielsson, F. , Stenström, L. , Arif, M. , Zhang, C. , Le, T. , Johansson, F. , Schutten, R. , Bäckström, A. , Axelsson, U. , Thul, P. , Cho, N. H. , Carja, O. , Uhlén, M. , Mardinoglu, A. , Stadler, C. , Lindskog, C. , … Lundberg, E. (2021). Spatiotemporal dissection of the cell cycle with single‐cell proteogenomics. Nature, 590, 649–654. 33627808 10.1038/s41586-021-03232-9

[117] Gray, G. K. , Li, C. M.‐C. , Rosenbluth, J. M. , Selfors, L. M. , Girnius, N. , Lin, J.‐R. , Schackmann, R. C. J. , Goh, W. L. , Moore, K. , Shapiro, H. K. , Mei, S. , D'andrea, K. , Nathanson, K. L. , Sorger, P. K. , Santagata, S. , Regev, A. , Garber, J. E. , Dillon, D. A. , Brugge‐C, J. S. , … Brugge, J. S. (2022). A human breast atlas integrating single‐cell proteomics and transcriptomics. Developmental Cell, 57, 1400–1420.e7. 35617956 10.1016/j.devcel.2022.05.003 PMC9202341

[118] Tracey, L. J. , An, Y. , & Justice, M. J. (2021). CyTOF: An emerging technology for single‐cell proteomics in the mouse. Current Protocols, 1, e118. 33887117 10.1002/cpz1.118

